# The protective effects of shikonin on hepatic ischemia/reperfusion injury are mediated by the activation of the PI3K/Akt pathway

**DOI:** 10.1038/srep44785

**Published:** 2017-03-21

**Authors:** Tong Liu, QingHui Zhang, Wenhui Mo, Qiang Yu, Shizan Xu, Jingjing Li, Sainan Li, Jiao Feng, Liwei Wu, Xiya Lu, Rong Zhang, Linqiang Li, Keran Cheng, Yuqing Zhou, Shunfeng Zhou, Rui Kong, Fan Wang, Weiqi Dai, Kan Chen, Yujing Xia, Jie Lu, Yingqun Zhou, Yan Zhao, Chuanyong Guo

**Affiliations:** 1 Department of Gastroenterology, Shanghai Tenth People’s Hospital, Tongji University School of Medicine, Shanghai 200072, China; 2 Department of Clinical Laboratory, Kunshan First People’s Hospital Affiliated to Jiangsu University, 215300, Kunshan, JiangSu, China; 3 Department of Gastroenterology, Minhang Hospital, Shanghai Medical School of Fudan University, Shanghai, 201100, China; 4 Department of Gastroenterology, Shanghai Tenth Hospital, School of Clinical Medicine of Nanjing Medical University, Shanghai 200072, China; 5 The School of Medicine of Soochow University, Suzhou 215006, China

## Abstract

Hepatic ischemia/reperfusion (I/R) injury, which can result in severe liver injury and dysfunction, occurs in a variety of conditions such as liver transplantation, shock, and trauma. Cell death in hepatic I/R injury has been linked to apoptosis and autophagy. Shikonin plays a significant protective role in ischemia/reperfusion injury. The purpose of the present study was to investigate the protective effect of shikonin on hepatic I/R injury and explore the underlying mechanism. Mice were subjected to segmental (70%) hepatic warm ischemia to induce hepatic I/R injury. Two doses of shikonin (7.5 and 12.5 mg/kg) were administered 2 h before surgery. Balb/c mice were randomly divided into four groups: normal control, I/R, and shikonin preconditioning at two doses (7.5 and 12.5 mg/kg). The serum and liver tissues were collected at three time points (3, 6, and 24 h). Shikonin significantly reduced serum AST and ALT levels and improved pathological features. Shikonin affected the expression of Bcl-2, Bax, caspase 3, caspase 9, Beclin-1, and LC3, and upregulated PI3K and p-Akt compared with the levels in the I/R group. Shikonin attenuated hepatic I/R injury by inhibiting apoptosis and autophagy through a mechanism involving the activation of PI3K/Akt signaling.

Hepatic ischemia-reperfusion (I/R) injury is a complex pathological process that occurs in association with liver transplantation, shock, trauma, and resection surgery, where the blood supply to the liver is temporarily interrupted^1^,^2^. It contributes to severe liver injury and dysfunction of the liver^3^ Hepatic I/R injury leads to the upregulation of inflammatory cytokines, such as tumor necrosis factor (TNF)-α, interleukin (IL)-1β, and IL-6^4^,^5^,^6^. Hepatic I/R injury is a common clinical occurrence that threatens the health of patients, underscoring the need to identify effective measures to protect against I/R injury.

Several signaling pathways are associated with hepatic I/R injury. Recent studies showed that activated phosphorylated Akt (p-Akt) significantly ameliorated I/R injury to the liver and other organs^7^,^8^,^9^. Apoptosis, also named type I programmed cell death, is closely associated with hepatic I/R injury^10^,^11^. Activation of PI3K/Akt signaling enhances anti-apoptotic Bcl-2 protein expression and protects cells against apoptosis^12^,^13^. The Bcl-2 family includes pro-apoptotic proteins, such as Bax and Bad, and anti-apoptotic proteins, such as Bcl-2 and Bcl-xl. The balance between Bcl-2 and Bax determines cell survival and death after injury^14^,^15^,^16^,^17^,^18^,^19^.

Autophagy, as a newly identified type of cell death, has attracted scientists’ attention. Autophagy is characterized by the formation of autophagosomes and autolysosomes and is an intracellular degradation process targeting impaired and damaged organelles^20^. Autophagy, to some degree, has a protective effect by recycling cell components under conditions of stress such as harsh environments^21^,^22^,^23^. However, beyond this range, autophagy will result in cell death, especially under conditions of sustained hypoxia, starvation, and inflammation^24^,^25^. Therefore, autophagy is considered to be a double-edged sword. The regulation of autophagy involves various genes, including Beclin-1, LC3, and P62^26^,^27^,^28^,^29^. Previous study reported that the inhibition of autophagy significantly attenuated hepatic I/R injury^10^. However, the underlying mechanism associated with apoptosis and autophagy in hepatic I/R injury remains uncertain and this issue needs further study.

To interfere with the process of cell death in hepatic I/R injury, we attempted to identify a new drug that could significantly ameliorate hepatic I/R injury. Shikonin, extracted from the root of *Lithospermum erythrorhizon*, possesses a variety of biological properties, including anti-inflammatory and anticancer effects^30^,^31^,^32^. The effects of shikonin on I/R injury have been investigated by scientists worldwide in recent years. Wang *et al*. reported that shikonin could prevent cerebral I/R injury in mice through its antioxidant activity^33^. Wang and his colleagues demonstrated that shikonin could significantly protect the brain against I/R injury by regulating inflammatory responses and improving blood-brain barrier (BBB) permeability^34^. However, the effects of shikonin on hepatic I/R injury remain unclear. Therefore, in the present study, we investigated the effects of shikonin on hepatic I/R injury and explored the underlying mechanisms. We hypothesized that shikonin could attenuate hepatic I/R injury by decreasing the levels of proinflammatory cytokines and reducing hepatic apoptosis and autophagy, which may be partly associated with the activation of the PI3K/Akt pathway.

## Results

### Shikonin at two doses and 2% DMSO had no effect on liver function

**Figure 1:**
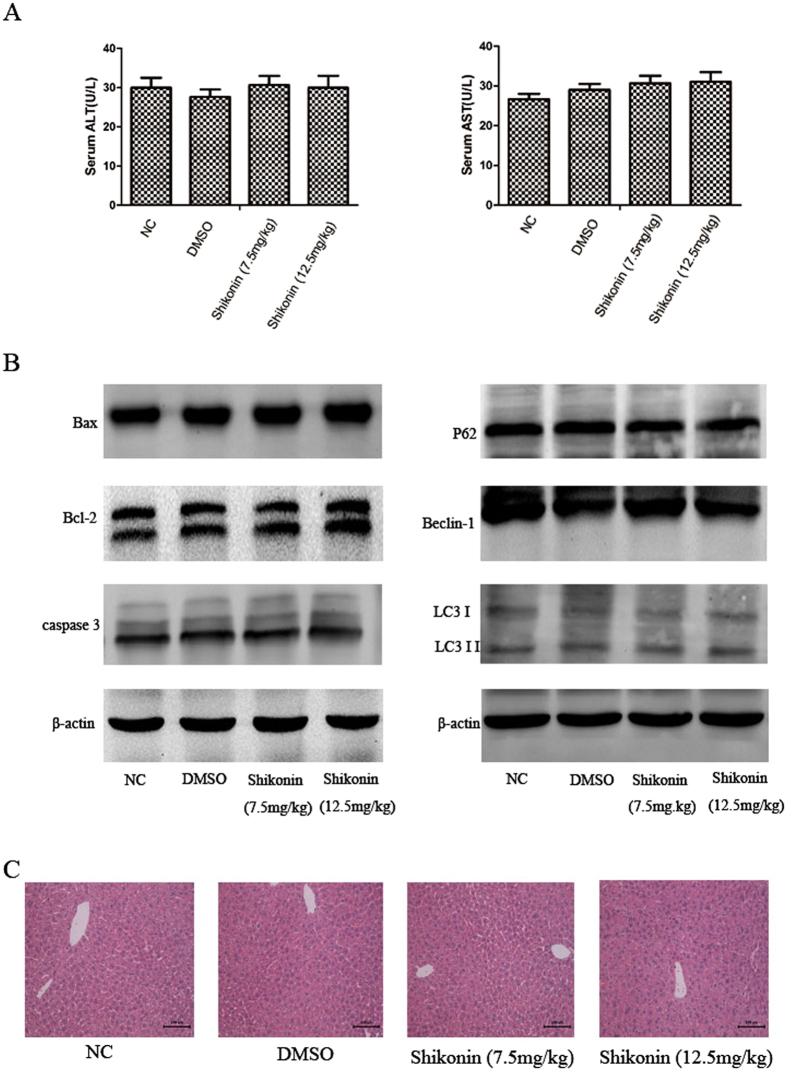
Shikonin at two doses and 2% DMSO had no injurious effects on liver function. (**A**) Serum AST and ALT levels were expressed as the mean ± SD (n = 6, *P* > 0.05). (**B**) The protein expression of Bcl-2, Bax, caspase 3, P62, Beclin-1, and LC3 was assessed by western blotting. The experiments were repeated three times. (**C**) Representative hematoxylin-and-eosin (HE) stained sections of liver tissues. Original magnification, ×200.

To determine whether shikonin and 2% DMSO affected liver function, the effects of two doses of shikonin (7.5 and 12.5 mg/kg) and 2% DMSO on liver enzymes and the expression of apoptosis and autophagy related proteins were examined. As shown in Fig. 1A, no differences in serum ALT and AST were detected between the DMSO group and the two shikonin groups and the normal control group. Figure 1B shows that there were no statistically significant differences in the expressions of Bax, Bcl-2, caspase 3, P62, Beclin-1, and LC3 among the four groups. Figure 1C shows no obvious necrosis in the four H&E stained images.

### Shikonin pretreatment ameliorates hepatic I/R injury in mice

**Figure 2:**
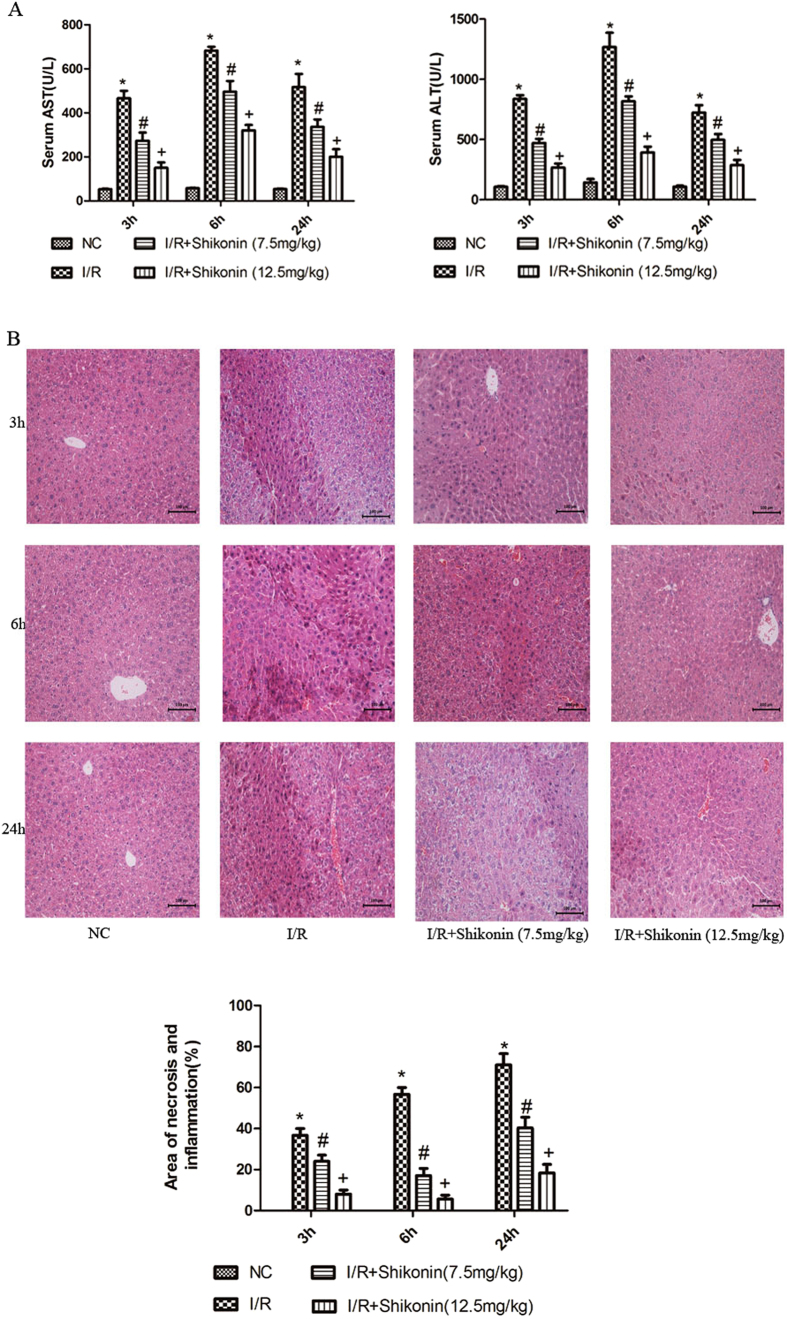
Pretreatment with shikonin ameliorated hepatic I/R injury. (**A**) Serum AST and ALT levels were expressed as the mean ± SD (n = 6, *P < 0.05 for I/R versus NC, ^#^*P* < 0.05 for I/R + Shikonin [7.5 mg/kg] versus I/R, ^+^*P* < 0.05 for I/R + Shikonin [12.5 mg/kg] versus I/R). (**B**) The necrotic area stained with HE was analyzed with Image-Pro Plus 6.0 (magnification, ×200). The results showed statistically significant differences (n = 6, *P < 0.05 for I/R versus NC, ^#^*P* < 0.05 for I/R + Shikonin [7.5 mg/kg] versus I/R, ^+^*P* < 0.05 for I/R + Shikonin [12.5 mg/kg] versus I/R).

After segmental (70%) hepatic warm ischemia for 45 min, the serum levels of AST and ALT were significantly increased at 3, 6, and 24 h compared with those in the normal control group, as shown in Fig. 2A, with the strongest effect at 6 h after hepatic I/R injury. However, shikonin pretreatment remarkably reduced the levels of AST and ALT at both doses (*P* < 0.05). Figure 2B shows the same trends in H&E staining. Abundant necrotic areas were observed in the I/R group, while the shikonin pretreatment groups showed minor necrotic areas at three time points, especially at 6 h. The results indicated that shikonin pretreatment dramatically ameliorated hepatic necrosis (*P* < 0.05). Image-Pro Plus software revealed statistically significant differences between the four groups. These results indicated that shikonin pretreatment effectively ameliorated hepatic I/R injury in mice.

### Shikonin pretreatment reduced the levels of inflammatory cytokines in hepatic I/R injury

**Figure 3:**
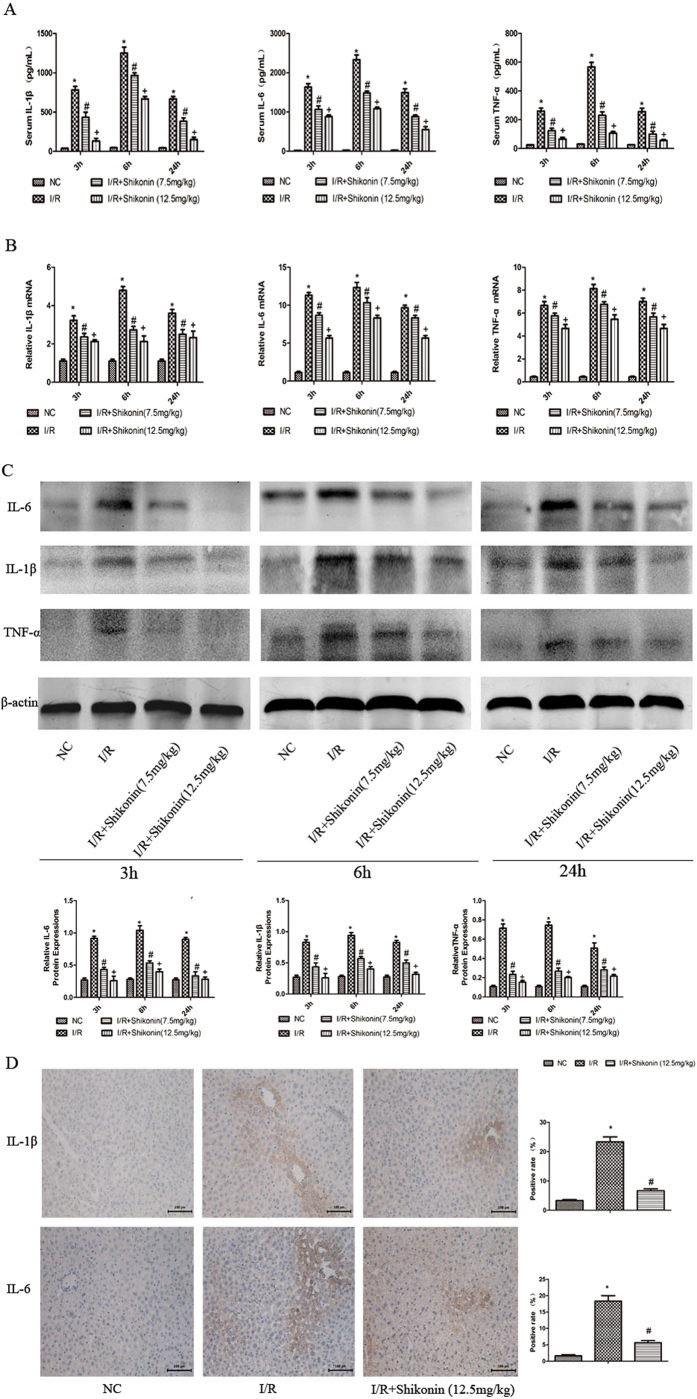
Shikonin reduced the release of IL-1β, TNF-α, and IL-6. (**A**) The serum levels of IL-1β, TNF-α, and IL-6 are shown as the mean ± SD (n = 6, *P < 0.05 for I/R versus NC, ^#^*P* < 0.05 for I/R + Shikonin [7.5 mg/kg] versus I/R, ^+^***P < ***0.05 for I/R + Shikonin [12.5 mg/kg] versus I/R). (**B**) The mRNA expression of IL-1β, TNF-α, and IL-6 was assessed by RT-PCR. The experiments were repeated three times and the data are shown as the mean ± SD (n = 6, *P < 0.05 for I/R versus NC, ^#^*P* < 0.05 for I/R + Shikonin [7.5 mg/kg] versus I/R, ^+^*P* < 0.05 for I/R + Shikonin [12.5 mg/kg] versus I/R). (**C**) The protein expression of IL-1β, TNF-α, and IL-6 was determined by western blotting and the gray values were calculated (n = 6, *P < 0.05 for I/R versus NC, ^#^*P* < 0.05 for I/R + Shikonin [7.5 mg/kg] versus I/R, ^+^*P* < 0.05 for I/R + Shikonin [12.5 mg/kg] versus I/R). (**D**) Representative immunohistochemical staining (×200) showing the expression of IL-1β and IL-6 at 6 h. The ratio of brown areas to the total area was analyzed with Image-Pro Plus 6.0 (n = 6, *P < 0.05 for I/R versus NC, ^#^*P* < 0.05 for I/R + Shikonin versus I/R).

The release of many inflammatory cytokines, including IL-1β, TNF-α, and IL-6, is associated with the occurrence and development of hepatic I/R injury^6^. Figure 3A shows that the serum levels of IL-1β, TNF-α, and IL-6 were increased in the I/R group compared with those in the normal control group at three time points, with a peak at 6 h after I/R injury. Shikonin pretreatment significantly reduced the levels of inflammatory cytokines, and both doses of shikonin were effective. Real-time PCR was used to determine the mRNA expression of these inflammatory factors. As shown in Fig. 3B, compared with the I/R group, shikonin pretreatment dramatically reduced the mRNA expression of IL-1β, TNF-α, and IL-6 at each time point. To confirm our results, we used western blotting to detect the protein expression of these cytokines. We found that the protein expression of IL-1β, TNF-α, and IL-6 was significantly increased in the I/R group at the three time points, while the protein expression of these cytokines decreased in both shikonin pretreatment groups (Fig. 3C). These results were consistent with the mRNA expression of IL-1β, TNF-α, and IL-6. To investigate further, immunohistochemical staining was used to determine the expression of inflammatory cytokines in the four groups (Fig. 3D). These results provided strong evidence that shikonin pretreatment could significantly reduce the release of IL-1β, TNF-α, and IL-6 in hepatic I/R injury in mice.

### Shikonin attenuated hepatocyte apoptosis in hepatic I/R injury

**Figure 4:**
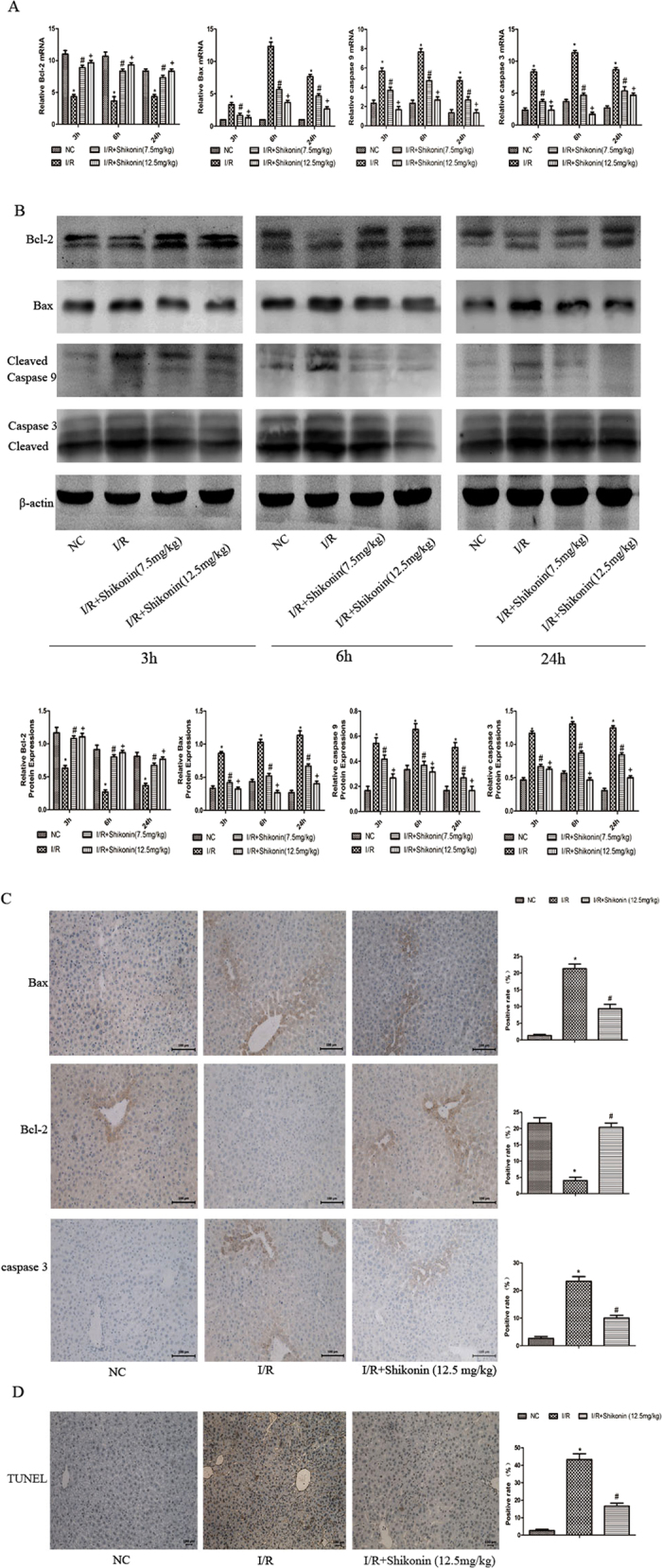
Shikonin reduced apoptosis in hepatic I/R injury. (**A**) The mRNA expression of Bcl-2, Bax, caspase 9, and caspase 3 was measured by RT-PCR and expressed as the mean ± SD (n = 6, *P < 0.05 for I/R versus NC, ^#^*P* < 0.05 for I/R + Shikonin [7.5 mg/kg] versus I/R, ^+^*P* < 0.05 for I/R + Shikonin [12.5 mg/kg] versus I/R). (**B**) The protein expression of Bcl-2, Bax, caspase 9, and caspase 3 was detected by western blotting and the gray values were calculated (n = 6, *P < 0.05 for I/R versus NC, ^#^*P* < 0.05 for I/R + Shikonin [7.5 mg/kg] versus I/R, ^+^*P* < 0.05 for I/R + Shikonin [12.5 mg/kg] versus I/R). (**C**) Immunohistochemical staining showing the expression of Bcl-2, Bax, and caspase 3 at 6 h. The ratio of brown areas to the total area was analyzed (n = 6, *P < 0.05 for I/R versus NC, ^#^*P* < 0.05 for I/R + Shikonin versus I/R). (**D**) TUNEL staining (×200) of liver tissues at 6 h to detect apoptotic cell (n = 6, *P < 0.05 for I/R versus NC, ^#^*P* < 0.05 for I/R + Shikonin versus I/R).

Bcl-2, Bax, caspase 9, and caspase 3 are important markers of apoptosis. Bcl-2 is an antiapoptotic protein, while Bax, caspase 9, and caspase 3 are proapoptotic proteins. Real-time PCR and western blotting were used to investigate the expression of markers of apoptosis at the mRNA and protein levels, respectively (Fig. 4A and B). The results showed that Bcl-2 was downregulated in the I/R group and upregulated in both shikonin groups at all three time points. However, Bax, caspase 9, and caspase 3 were upregulated in the I/R group and downregulated in the shikonin treatment groups at each time point. As shown in Fig. 4C, the results of immunohistochemical staining were consistent with the results of real-time PCR and western blotting. Figure 4D shows the results of TUNEL staining, which demonstrated that abundant apoptotic cells were seen in the I/R group and few apoptotic cells were detected in the shikonin treatment group. The histogram indicated statistical significance (*P* < 0.05).

### Shikonin inhibited hepatocyte autophagy in hepatic I/R injury

**Figure 5:**
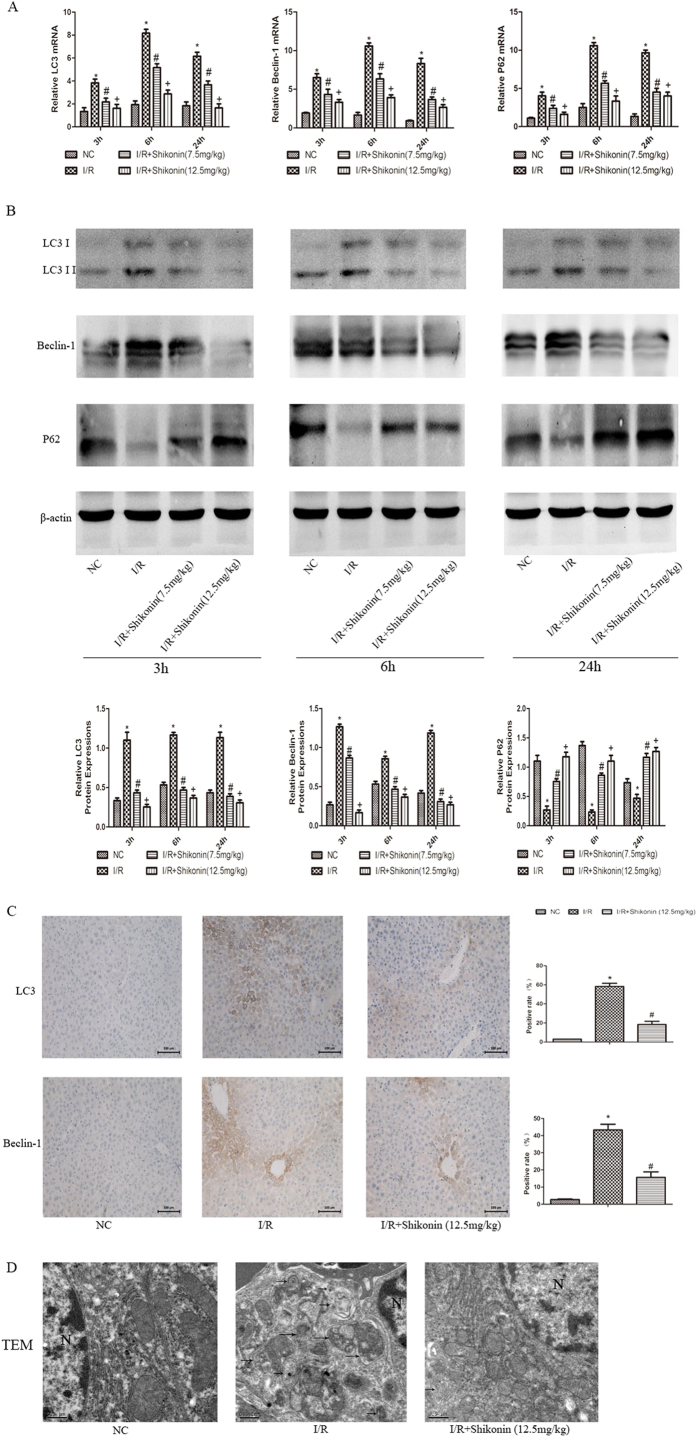
Shikonin inhibited autophagy in hepatic I/R injury. (**A**) The mRNA expression of LC3, Beclin-1, and P62 was measured by RT-PCR and expressed as the mean ± SD (n = 6, *P < 0.05 for I/R versus NC, ^#^*P* < 0.05 for I/R + Shikonin [7.5 mg/kg] versus I/R, ^+^*P* < 0.05 for I/R + Shikonin [12.5 mg/kg] versus I/R). (**B**) The protein levels of LC3, Beclin-1, and P62 were detected by western blotting and the gray values were calculated (n = 6, *P < 0.05 for I/R versus NC, ^#^*P* < 0.05 for I/R + Shikonin [7.5 mg/kg] versus I/R, ^+^*P* < 0.05 for I/R + Shikonin [12.5 mg/kg] versus I/R). (**C**) Representative immunohistochemical staining (×200) showing the expression of LC3 and Beclin-1 at 6 h. The ratio of brown areas to the total area was analyzed with Image-Pro Plus (n = 6, *P < 0.05 for I/R versus NC, ^#^*P* < 0.05 for I/R + Shikonin versus I/R). (**D**) Arrows indicate autophagosomes detected by transmission electron microscopy (TEM). Magnification: 20,000×.

Beclin-1, LC3, and P62 play significant roles in the occurrence and development of autophagy. We used PCR and western blotting to determine the expression of these markers at the mRNA and protein levels (Fig. 5A and B). Beclin-1 and LC3 were significantly upregulated in the I/R group, while P62 was downregulated in the I/R group. The expression of Beclin-1, LC3, and P62 was dramatically inhibited by shikonin treatment at 3, 6 and 24 h. As shown in Fig. 5C, the results of immunohistochemistry staining were consistent with those of PCR and western blotting. To investigate further, transmission electron microscopy was used to detect autophagosomes in hepatocytes at 6 h. Figure 5D shows that lysosomes and autophagosomes were clearly increased in the I/R group, while few lysosomes and autophagosomes were detected in the shikonin pretreatment group. In conclusion, these results demonstrated that shikonin inhibited hepatocyte autophagy and protected the liver from I/R injury.

### Shikonin attenuated hepatic apoptosis and autophagy through the activation of the PI3K/Akt pathway in hepatic I/R injury

**Figure 6:**
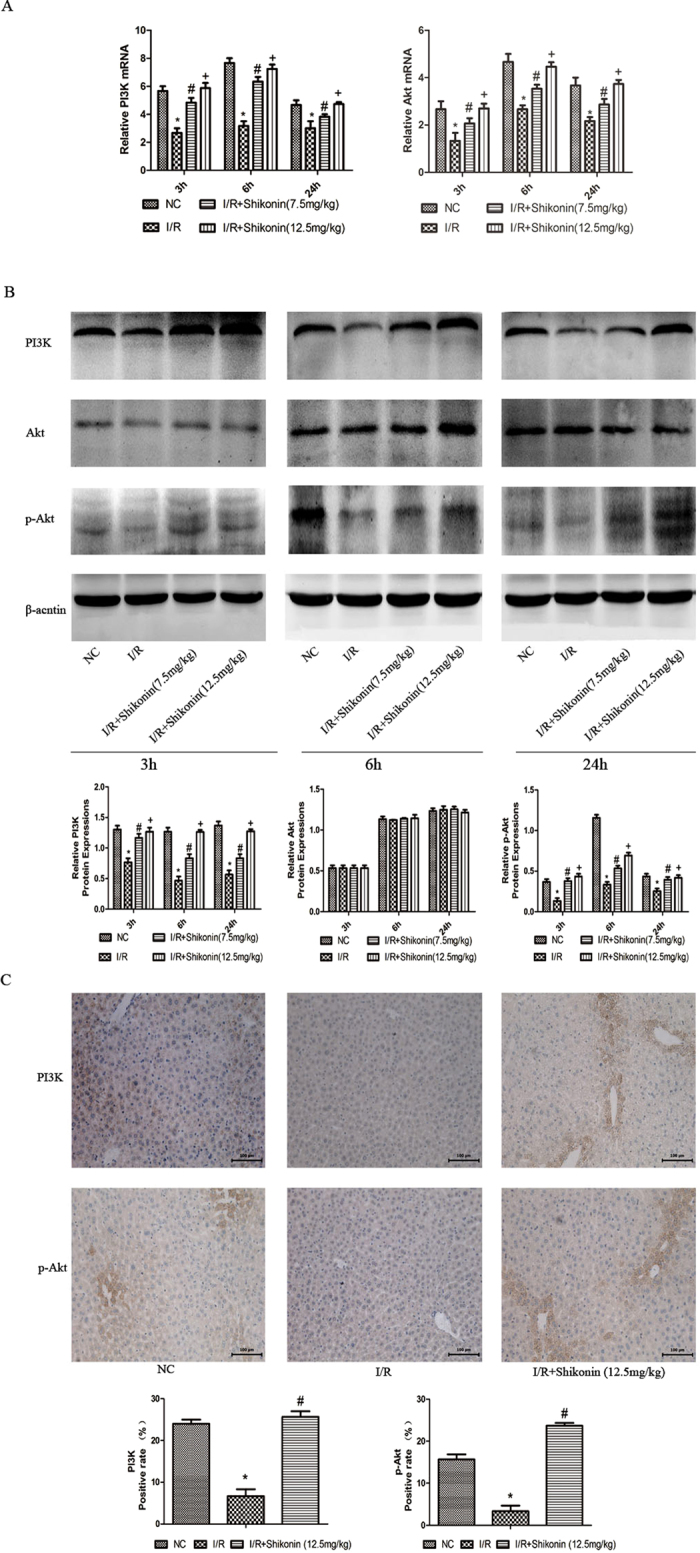
Shikonin ameliorated hepatic I/R injury by activating the PI3K/Akt pathway. (**A**) The mRNA expression of PI3K and Akt was detected by RT-PCR and the data are expressed as the mean ± SD (n = 6, *P < 0.05 for I/R versus NC, ^#^*P* < 0.05 for I/R + Shikonin [7.5 mg/kg] versus I/R, ^+^*P* < 0.05 for I/R + Shikonin [12.5 mg/kg] versus I/R). (**B**) The protein expression of PI3K, Akt, and p-Akt was detected by western blotting and the gray values were calculated (n = 6, *P < 0.05 for I/R versus NC, ^#^*P* < 0.05 for I/R + Shikonin [7.5 mg/kg] versus I/R, ^+^*P* < 0.05 for I/R + Shikonin [12.5 mg/kg] versus I/R). (**C**) Immunohistochemical staining (×200) showing the expression of PI3K and p-Akt at 6 h. The ratio of brown areas to the total area was analyzed with Image-Pro Plus (n = 6, *P < 0.05 for I/R versus NC, ^#^*P* < 0.05 for I/R + Shikonin versus I/R).

The above results demonstrated that shikonin could attenuate apoptosis and autophagy in hepatic I/R injury. However, the underlying mechanism remained unclear. The PI3K/Akt pathway plays an important role in cellular survival and apoptosis. Therefore, we evaluated whether shikonin protected liver tissues against I/R injury through the activation of the PI3K/Akt pathway. Real-time PCR was used to investigate the mRNA levels of PI3K and Akt. As shown in Fig. 6A, the mRNA levels of PI3K and Akt were significantly increased at both doses of shikonin compared with the I/R group at three time points. The protein levels of PI3K, Akt, and p-Akt are shown in Fig. 6B. The protein levels of PI3K and p-Akt were clearly increased in the shikonin-pretreated groups compared with the I/R group at three time points. However, the protein level of Akt did not differ between the four groups at the three time points. The results of immunohistochemical staining of PI3K and p-Akt were consistent with those of PCR and western blotting (Fig. 6C). Taken together, these results provided strong evidence that shikonin attenuated apoptosis and autophagy partly by activating the PI3K/Akt pathway.

## Discussion

Hepatic ischemia/reperfusion (I/R) injury is a complex pathological process associated with liver transplantation, shock, and trauma. The process of hepatic I/R injury is a serious threat to human health. Recent report reported that intestinal ischemia/reperfusion could also lead to acute lung injury and increase the mortality in mice^35^. However, no effective treatments have been identified for clinical use. The identification of effective instruments and drugs is essential. Recent research has demonstrated that tea polyphenols derived from tea extracts can protect against hepatic I/R injury in mice^36^. Therefore, we attempted to identify new natural agents like tea polyphenols that could ameliorate hepatic I/R injury. Shikonin, a promising anti-inflammation drug, has attracted worldwide attention among scientists.

Recent studies showed that shikonin exerts protective effects in brain I/R injury^33^,^34^. However, the underlying mechanism in hepatic I/R injury remains unknown. Therefore, we established a model of hepatic I/R injury to investigate the protective effects of shikonin and the mechanisms involved. Several studies showed that there is a close relationship between inflammatory responses and hepatic I/R injury^10^,^37^. Therefore, we investigated the expression of IL-1β, TNF-α, and IL-6 at the mRNA and protein levels. Our results showed that pretreatment with shikonin significantly decreased the levels of these inflammatory cytokines compared with those in the I/R group, and both doses of shikonin (7.5 and 12.5 mg/kg) were effective, with a stronger effect obtained with 12.5 mg/kg (Fig. 3B and C). These results were consistent with the serum AST and ALT levels and pathological changes. The serum levels of AST and ALT were markedly reduced in response to both doses of shikonin. Extensive necrosis of liver tissues was observed in the I/R group, which was clearly ameliorated in response to both doses of shikonin at all three time points, particularly at 6 h (Fig. 2A and B). Our results demonstrated that shikonin pretreatment suppressed the release of inflammatory cytokines, including IL-1β, TNF-α, and IL-6, and attenuated serum liver enzyme levels and pathological changes in hepatic I/R injury.

Several pathways have been reported to be associated with hepatic I/R injury, including ROS/MAPK pathway, ROS/JNK/Bcl-2 pathway and HMGB1/TLR4/NF-kappab pathway. The PI3K/Akt pathway, a well-known cell survival pathway, plays critical roles in the regulation of cell proliferation and cell apoptosis^38^. Moreover, the PI3K/Akt signaling pathway exerts a strong protective effect on I/R injury through the inhibition of apoptosis^39^,^40^. A recent study showed that shikonin attenuated chondrocyte apoptosis by upregulating the PI3K/Akt pathway^41^. Huang and his colleagues found that shikonin protected oxidized low-density lipoprotein (oxLDL)-induced endothelial damage by activating the PI3K/Akt pathway^42^. However, the underlying mechanism of shikonin in hepatic I/R injury remains unclear. We therefore investigated whether shikonin protected liver tissues in I/R injury through the activation of the PI3K/Akt pathway. RT-PCR, western blotting, and immunohistochemistry were used to examine the expression of PI3K and p-Akt. Our results demonstrated that shikonin pretreatment upregulated PI3K and p-Akt, which were significantly decreased in the I/R group, indicating that shikonin ameliorated hepatic I/R injury through the activation of PI3K and p-Akt.

**Figure 7:**
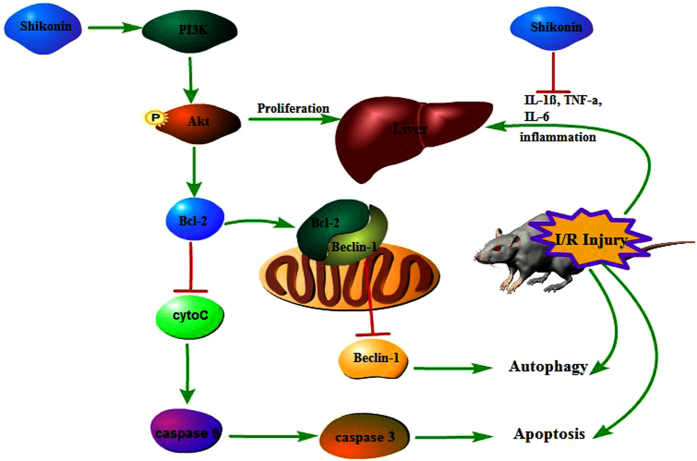
Mechanism of the protective effects of shikonin on hepatic I/R injury. Shikonin possesses anti-inflammatory effect and decreases the levels of pro-inflammatory cytokines such as IL-1β, IL-6, and TNF-α. Shikonin activates PI3K and promotes the phosphorylation of its downstream Akt, leading to the proliferation of injured hepatocytes. Phosphorylated Akt promotes the expression of Bcl-2, which inhibits the release of cytoC and down-regulates the expression of caspase 3 and caspase 9, leading to the inhibition of apoptosis. The upregulation of Bcl-2 inhibits the dissociation of Beclin1 from Bcl-2 and results in the reduction of Beclin-1, leading to the inhibition of autophagy.

The pathophysiological mechanism underlying the effect of shikonin on reducing apoptosis through the activation of PI3K and p-Akt is not clear. Liu *et al*. reported that dehydroepiandrosterone (DHEA) attenuates vascular endothelial cell apoptosis via the activation of PI3K/Akt and the enhancement of Bcl-2 expression^12^. Kumar *et al*. found that vascular endothelial growth factor (VEGF)-treated human dermal microvascular endothelial cells (HDMECs) are protected from gamma-irradiation-induced apoptosis through the PI3K-Akt-Bcl-2 pathway^13^. Therefore, we investigated whether shikonin reduced hepatic I/R injury by activating PI3K/Akt and increasing the expression of Bcl-2. To investigate the anti-apoptotic mechanism of shikonin, we measured the expression of Bcl-2 and Bax. Bcl-2 and Bax belong to the Bcl-2 family; Bcl-2 is an anti-apoptotic protein and Bax is a pro-apoptotic protein. Bcl-2 inhibits apoptosis by reducing the release of cytochrome c (cyto C)^43^, leading to a reduction in the release of caspase 9 and caspase 3. Therefore, caspase-mediated apoptosis is inhibited (Fig. 7). Our results showed that I/R injury upregulated Bax, caspase 9, and caspase 3, and downregulated Bcl-2, while shikonin significantly upregulated the expression of Bcl-2 and reduced the expression of Bax, caspase 9, and caspase 3 (Fig. 4). These results provided strong evidence that shikonin reduced cell apoptosis in hepatic I/R injury via the activation of PI3K/Akt and the enhancement of Bcl-2 expression.

The Bcl-2/Beclin-1 complex plays a critical role in the regulation of apoptosis and autophagy^44^. Shikonin upregulated the expression of PI3K and p-Akt, thereby upregulating Bcl-2 protein expression through the PI3K/Akt pathway. A recent study showed that the upregulation of Bcl-2 could inhibit the dissociation of Beclin1 from Bcl-2^45^. In other words, the upregulation of Bcl-2 could enhance the formation of a Bcl-2/Beclin-1 complex, leading to a reduction of Beclin-1 expression and the inhibition of autophagy. Beclin-1, LC3, and P62 are considered markers of autophagy. We used RT-PCR and western blotting to detect the expression of these markers. Our results showed that both doses of shikonin significantly decreased the expression of LC3 and Beclin-1 and increased the expression of P62 compared with the results in the I/R group. The results of immunohistochemistry and transmission electron microscopy were consistent with those of RT-PCR and western blotting. The above results demonstrated that shikonin suppressed autophagy by activating the PI3K/Akt pathway, thus preventing hepatic I/R injury.

Hepatic I/R injury involves various complex and multifactorial mechanisms and these mechanisms need to be further explored. In our present study, we investigated the preventive effect of shikonin on hepatic I/R injury. However, we did not explore the therapeutic potential of shikonin. We will further investigate the therapeutic effect and mechanism of shikonin on hepatic I/R injury in our future studies.

## Materials and Methods

### Reagents

Shikonin and DMSO were purchased from Sigma-Aldrich (Saint Louis, MO, USA). The antibodies used in the study were purchased from Cell Signaling Technology (Danvers, MA, USA), including antibodies against TNF-α, IL-1β, IL-6, Bax, Bcl-2, caspase3, caspase9, LC3, Beclin-1, and P62. PCR kits were purchased from Takara (Takara Biotechnology, Dalian, China). The kits for aspartate aminotransferase (AST) and alanine aminotransferase (ALT) were purchased from Nanjing Jiancheng Bioengineering Institute (Jiancheng Biotech, China).

### Animals

Male Balb/c mice (6–8 weeks old, 22 ± 2 g) were purchased from Shanghai Laboratory Animal Co., Ltd., Shanghai, China. Mice were raised in plastic cages, which were maintained at 24 ± 2 °C. The mice had free access to water and food. All experimental designs were approved by the Animal Care and Use Committee of The Tenth People’s Hospital of Shanghai, Tongji University. This research was approved by the Science and Technology Commission of Shanghai Municipality (ID: SYXK 2011-0111).

### Preliminary study

The study included 72 mice randomly assigned to four groups: normal control (treated with saline only), DMSO (treated with 2% DMSO), low dose (treated with shikonin at 7.5 mg/kg), and high dose (treated with shikonin at 12.5 mg/kg). Shikonin was diluted in 2% DMSO. Six mice were randomly selected and killed. The serum and liver tissues were immediately collected and used to detect serum AST and ALT, the expression of apoptosis and autophagy marker proteins, and pathological changes.

### Model establishment and experimental design

A mouse model of segmental (70%) hepatic warm ischemia was established using a previously reported method^46^. Mice were fasted for 24 h and placed on a sterile table after receiving an intraperitoneal injection of 1.25% Nembutal (Sigma-Aldrich). All mice underwent midline laparotomy. All structures in the portal triad were blocked for 45 min with a metal microvascular clamp. After 45 min, the clamps were loosened to begin liver reperfusion. After reperfusion, the abdominal incision was closed with surgical thread and the mice were placed in a warm environment until they were awake. A total of 72 mice were randomly divided into four groups as follows:

Group I, normal control group (n = 18): mice were intraperitoneally injected with saline 2 h before laparotomy without I/R.

Group II, I/R group (n = 18): mice were intraperitoneally injected with saline 2 h before laparotomy with I/R for 45 min.

Group III, low dose group (n = 18): mice were intraperitoneally injected with 7.5 mg/kg shikonin 2 h before laparotomy with I/R for 45 min.

Group IV, high dose group (n = 18): mice were intraperitoneally injected with 12.5 mg/kg shikonin 2 h before laparotomy with I/R for 45 min.

Six mice from each group were randomly selected and killed at 3, 6, and 24 h after hepatic I/R. All serum and liver tissues were immediately collected and stored for further experiments.

### Analysis of liver enzymes

The levels of AST and ALT were detected with a chemical analyzer (Olympus AU1000, Tokyo, Japan). The levels of serum IL-1β, TNF-α, and IL-6 were measured with ELISA (enzyme-linked immunosorbent assay) kits (R&D Systems, USA) following the manufacturer’s protocols.

### Histopathology

Tissues from the median and left lobes of the liver were collected and incubated in 4% paraformaldehyde for at least 24 h, then embedded in paraffin. Sections (4 μm thick) were stained with hematoxylin-eosin (H&E) and used to observe inflammation and tissue damage by light microscopy.

### Immunohistochemical staining

The prepared liver sections were heated at 67 °C for 45 min and then dewaxed in dimethylbenzene for 10 min. After dewaxing, the sections were rehydrated with a graded series of alcohol and pretreated with an antigen-retrieval technique consisting of heating in a water bath at 90 °C for 10 min and cooling for 5 min to recover the antigens. To block endogenous peroxidase activity, the samples were treated with 3% H_2_O_2_ for 15 min at 37 °C. Nonspecific binding was blocked using 5% bovine serum albumin (BSA) at room temperature for 20 min. The specimens were finally incubated overnight with antibodies against IL-1β (1:100), IL-6 (1:100), Bax (1:100), Bcl-2 (1:100), caspase 3 (1:100), LC3 (1:100), Beclin-1 (1:100), PI3K (1:100), and p-Akt (1:100). Then, the liver specimens were washed with phosphate-buffered saline (PBS) and immediately incubated with the secondary antibody (1:500 in PBS) for 30 min. Finally, antibody binding was analyzed using a diaminobenzidine (DAB) kit, and the liver specimens were observed using a light microscope. Image-Pro Plus 6.0 was used to calculate the ratios of brown staining areas to total areas.

### Western blot analysis

Liver tissues were recovered from −80 °C storage and immediately placed in liquid nitrogen. Then, the tissues were lysed with RIPA lysis buffer supplemented with protease inhibitors and phenylmethanesulfonyl fluoride. Protein concentration was detected with the bicinchoninic acid protein assay (Kaiji, China). Protein samples were incubated in 100 °C water for 10 min and separated by sodium dodecyl sulfate polyacrylamide gel electrophoresis with gels of different concentrations, followed by transfer to polyvinylidene difluoride (PVDF) membranes. Then, the membranes were blocked with 5% nonfat milk for 60 min and incubated overnight at 4 °C with primary antibodies against β-actin (1:1000), IL-1β (1:200), TNF-α (1:200), IL-6 (1:200), Bcl-2 (1:500), Bax (1:500), caspase 3 (1:500), caspase 9 (1:500), LC3 (1:500), Beclin-1 (1:1000), P62 (1:500), PI3K (1:500), Akt (1:500), and p-Akt (1:200). The following day, the membranes were washed three times for 10 min each with PBST and incubated with secondary antibody (1:2000) for 60 min at 37 °C. Finally, the membranes were washed three times for 10 min each with PBST in the dark, and the results were analyzed using the Odyssey Two-Color Infrared Laser Imaging System.

### TUNEL staining

After the liver sections were dewaxed, dehydrated, and rehydrated, TUNEL staining was performed according to the protocols of the TUNEL assay kit. Then, the liver sections were re-dyed with hematoxylin. TUNEL positive cells and hepatocytes were observed using a microscope.

### Transmission Electron Microscopy (TEM)

A small piece of the left lobe of the liver was placed in 4% glutaraldehyde and post-fixed in 1% osmium tetroxide for 1 h to observe autophagic vesicles. Finally, the cells were observed by TEM (JEOL, JEM 1230).

### RT-PCR and SYBR green real-time RT-PCR

RNA was extracted from frozen liver tissues stored at −80 °C using the TRIzol reagent (Takara, Shiga, Japan) and reverse transcribed into cDNA following the manufacturer’s instructions (Takara). SYBR green quantitative RT-PCR was used to detect the expression of target genes. The primers used for RT-PCR are listed in Table 1.

### Statistical Analysis

All the experiments were repeated at least three times. All results are expressed as the mean ± SD. One-way ANOVA was used to determine statistical differences between groups. In all comparisons, P < 0.05 was considered statistically significant. All statistical analyses were performed using SPSS 17.0. All methods were performed in accordance with the guidelines and regulations of Shanghai Tongji University.

## Additional Information

**How to cite this article:** Liu, T. *et al*. The protective effects of shikonin on hepatic ischemia/reperfusion injury are mediated by the activation of the PI3K/Akt pathway. *Sci. Rep.* **7**, 44785; doi: 10.1038/srep44785 (2017).

**Publisher's note:** Springer Nature remains neutral with regard to jurisdictional claims in published maps and institutional affiliations.

## Figures and Tables

**Table 1:** Nucleotide sequences of primers used for PCR.

Gene		Primer sequence (5′—3′)
IL-1β	Forward	CGATCGCGCAGGGGCTGGGCGG
	Reverse	AGGAACTGACGGTACTGATGGA
TNF-α	Forward	CAGGCGGTGCCTATGTCTC
	Reverse	CGATCACCCCGAAGTTCAGTAG
IL-6	Forward	CTGCAAGAGACTTCCATCCAG
	Reverse	AGTGGTATAGACAGGTCTGTTGG
Bcl-2	Forward	GCTACCGTCGTCGTGACTTCGC
	Reverse	CCCCACCGAACTCAAAGAAGG
Bax	Forward	AGACAGGGGCCTTTTTGCTAC
	Reverse	AATTCGCCGGAGACACTCG
Caspase 3	Forward	CTCGCTCTGGTACGGATGTG
	Reverse	TCCCATAAATGACCCCTTCATCA
Caspase 9	Forward	GGCTGTTAAACCCCTAGACCA
	Reverse	TGACGGGTCCAGCTTCACTA
Beclin-1	Forward	ATGGAGGGGTCTAAGGCGTC
	Reverse	TGGGCTGTGGTAAGTAATGGA
P62	Forward	GAGGCACCCCGAAACATGG
	Reverse	ACTTATAGCGAGTTCCCACCA
LC3	Forward	GACCGCTGTAAGGAGGTGC
	Reverse	AGAAGCCGAAGGTTTCTTGGG
PI3K	Forward	CACTCAGCCCATCTATTTCCAG
	Reverse	TCTTGGATCTTCACCTTCAGC
Akt	Forward	GACTGACACCAGGTATTTCGATGA
	Reverse	CTCCGCTCACTGTCCACACA
β-actin	Forward	GGCTGTATTCCCCTCCATCG
	Reverse	CCAGTTGGTAACAATGCCATGT
